# Helicobacter pylori infection, clarithromycin-resistant genes and CYP2C19 gene polymorphisms in asymptomatic school-age children in Baigou New Town, Baoding City, Hebei Province

**DOI:** 10.12669/pjms.41.2.9838

**Published:** 2025-02

**Authors:** Jie Zhang, Weicheng Yuan, Yutong Xing, Xinyu Ma, Fan Xuan

**Affiliations:** 1 Jie Zhang, Hebei Medical University, Shijiazhuang 050000, Hebei, China; 2 Weicheng Yuan, Hebei Medical University, Shijiazhuang 050000, Hebei, China; 3 Yutong Xing Department of Pediatrics, The Second Hospital of Hebei Medical University, Shijiazhuang 050000, Hebei, China; 4 Xinyu Ma Department of Pediatrics, The Second Hospital of Hebei Medical University, Shijiazhuang 050000, Hebei, China; 5 Fan Xuan Department of Pediatrics, The Second Hospital of Hebei Medical University, Shijiazhuang 050000, Hebei, China

**Keywords:** Clarithromycin resistance genes, CYP2C19 gene polymorphisms, Helicobacter pylori, School-age children

## Abstract

**Objective:**

To investigate the prevalence of Helicobacter pylori (Hp) infection, clarithromycin (CAM)-resistant genes and CYP2C19 gene polymorphisms in asymptomatic school-age children in Baigou New Town, Baoding City, Hebei Province, China.

**Methods:**

This retrospective study recruited 1,068 primary school students from Baigou New Town, Baoding City, Hebei Province, China, in November 2023 to screen for Hp infection and detect clarithromycin-resistant genes using stool samples. Additionally, blood samples were collected for CYP2C19 gene polymorphism analysis.

**Results:**

The stool antigen test for Hp yielded a positive rate of 18.4% (197/1,068). Among the 197 Hp-positive patients, 36.5% were resistant to CAM. The polymorphism analysis revealed that, in the 23S rRNA V region, the overall mutation rate was 62.5% for the A2143G site, 77.7% for T2182C, 50% for A2143G and T2182C, 2.7% for A2142C and 1.3% for A2142G. Moreover, the analysis classified the study participants into three CYP2C19 phenotypes (i.e., extensive, intermediate and poor metabolisers; extensive metabolisers (EM), intermediate metabolisers (IM) and poor metabolisers (PM), respectively), and the prevalence was 45.6%, 44.6% and 9.6% for EM, IM and PM, respectively.

**Conclusion:**

The local area showed a high CAM resistance, indicating that CAM is not preferred for Hp eradication. CYP2C19 gene polymorphism analysis revealed that the EM phenotype was highly prevalent. Therefore, a comprehensive analysis of CYP2C19 gene polymorphisms is recommended to identify ideal proton-pump inhibitors for Hp eradication.

## INTRODUCTION

Helicobacter pylori (Hp) infection is prevalent worldwide and associated with various systemic diseases, such as gastroduodenal disorders, inflammatory bowel disease, gastroesophageal reflux disease, liver cancer and cholecystitis.^1^ Studies have shown a decline in the prevalence of Hp infection in mainland China and a high prevalence in economically underdeveloped regions.^2^ Owing to the widespread use of antibiotics, Hp resistance is on the rise. Accordingly, in recent years, the eradication rate of Hp infection using the triple therapy of amoxicillin, clarithromycin (CAM) and proton-pump inhibitors (PPIs) has dropped to <80%.^3^

The failure of Hp eradication is attributed to CAM resistance and cytochrome P450 2C19 (CYP2C19) gene polymorphisms. Most studies on Hp resistance have shown a high CAM resistance rate, which is a significant factor contributing to decreased Hp eradication rates.^4^ Regarding CYP2C19 gene polymorphisms, Hp eradication failure was mainly due to the resultant poor response to PPI therapy.^5^ This study enrolled 1,068 students from two primary schools in Baigou New Town, Baoding City, Hebei Province, China, to determine the prevalence of Hp infection, CAM resistance and CYP2C19 gene polymorphisms in school-age children. Additionally, a questionnaire analysis was performed to investigate potential risk factors for Hp infection.

## METHODS

This retrospective study recruited 1,068 students from two primary schools in Baigou New Town, Baoding City, Hebei Province, China, in November 2023 as the study participants. The students were aged 7-12 years. There were 712 males and 356 females, with a mean age of 9.7 ± 1.5 and 9.8 ± 1.6 years, respectively.

### Ethics Committee Approval:

The study was approved by the Medical Ethics Committee of the Second Hospital of Hebei Medical University (no. 2023-R722; Dated: October 12, 2023). Informed consent forms were signed by the legal guardians of all enrolled children.

### Inclusion criteria:

- No use of antibiotics within the past month and acid-suppressive drugs within the past two weeks.
- Aged 7-12 years.
- Voluntary provision of stool and blood samples as required.

### Exclusion criteria:

- Severe cardiovascular, renal, hepatic or endocrine system diseases.
- Digestive symptoms (e.g., abdominal pain, nausea and vomiting).
- Absence of informed consent.

### Hp Detection:

A fresh stool sample was obtained from each student and kept in a dedicated collection box on the same day. The samples were stored at −4°C and transported to a laboratory for nucleic acid extraction. Real-time polymerase chain reaction was conducted to detect Hp- and CAM-resistant genes.

### CYP2C19 Gene Polymorphism Analysis:

Fasting blood samples (2 mL) were collected from all participants by a dedicated professional in the morning. Then, the samples were sent to the Precision Medicine Laboratory of Baoding Children’s Hospital using first-generation sequencing. Detection procedures were conducted by qualified professionals. Based on the detection results, the participants were classified as extensive metabolisers (EM), intermediate metabolisers (IM) and poor metabolisers (PM).

### Statistical analysis:

The SPSS25.0 software was used for statistical analysis. The confidence interval was 95%. Intergroup comparisons of categorical variables were examined utilising the chi-square (*χ*²) test or Fisher’s exact test. *P*< 0.05 indicated statistical significance.

## RESULTS

Among the 1,068 children included in this study, 197 (18.4%) tested positive for stool Hp antigen, including 127 males and 70 females, with an average age of 10.5 ± 1.5 years. Hp infection prevalence was lower in children aged ≤10 years than in those aged >10 years (*P* < 0.05) (Table-I). The 197 Hp-positive participants, 72 (36.5%) exhibited CAM resistance. The gene polymorphism analysis of the 23S rRNA V region performed on these 72 CAM-resistant children showed that the overall mutation rate was 62.5% (45/72) for the A2143G site, 77.7% (56/72) for T2182C, 50% (36/72) for A2143G and T2182C, 2.7% (2/72) for A2142C and 1.3% (1/72) for A2142G (Table-II). Among the 197 paediatric patients with CYP2C19 gene results, 90 (45.6%) were classified as EM, 88 (44.6%) as IM and 19 (9.6%) as PM (Table-III).

**Table-I:** Comparison of Hp infection among school-age children in different age groups.

Group	≤10 years	>10 years	χ²	p
Hp-positive cases	64	133	94.528	0.001
Total cases	670	398		

**Table-II:** Results of gene polymorphism analysis of the 23S rRNA V region in 72 CAM-resistant cases.

Phenotype	Mutations at A2143G (%)	Mutations at T2182C (%)	Mutations at A2143G and T2182C (%)	Mutations at A2142C (%)	Mutations at A2142G (%)
CAM-resistant cases	45 (62.5%)	56 (77.7%)	35 (50%)	2 (2.7%)	1 (1.3%)

**Table-III:** CYP2C19 gene polymorphisms.

Phenotype	EM (%)	IM (%)	PM (%)
CYP2C19 gene polymorphisms	90 (45.6%)	88 (44.6%)	19 (9.6%)

## DISCUSSION

Our study revealed an Hp infection rate of 18.4% among asymptomatic children in our region, indicating that the Hp infection rate among children is at a relatively high level, highlighting the importance of screening for Hp infection among children in the local community. Consistent with these findings, Hp infection is prevalent among children in China. Previous reports from Beijing, Chengdu and Guangzhou revealed an Hp infection rate of 6.8% in asymptomatic children and that prevalence increases with age.^6^ Specifically, the Hp infection rate among children aged 7-12 years was reported to be 24.1%.^7^

Our study results showed that in the local area, the resistance rate for CAM was 36.5% among the asymptomatic Hp-infected children, demonstrating a high CAM resistance in paediatric cases of Hp infection. With the exacerbation of antibiotic resistance, particularly the increasing CAM resistance rate, the triple therapy of amoxicillin, CAM and PPIs has become less effective in Hp eradication in children.^8^,^9^ In the United States, the CAM resistance rate was up to 32.3% in Hp infection cases^10^, whereas in Europe, it reached 45% among children with Hp infection.^11^ In asymptomatic children in northern Peru, the CAM resistance rate was reported to be 79.6%.^12^ In China, the resistance rates for CAM among children were 47.3% in Chongqing and 20.6% in Hangzhou.^13^,^14^

The present study revealed an overall mutation rate of 62.5% for the A2143G site, 77.7% for T2182C and 50% for A2143G and T2182C. This indicates that in our region, Hp resistance to CAM is mainly associated with mutations at the A2143G and T2182C sites in the 23S rRNA gene. CAM is the most commonly used antibiotic in Hp eradication, and treatment failure is primarily caused by the increasing CAM resistance rate. This resistance is associated with 23S rRNA gene mutations.^15^ Among Hp-resistant strains isolated from Chinese children, mutations are most frequently observed at the A2143G site in the CAM 23S rRNA gene.^16^

Our study results show that in the local area, the prevalence was 45.6%, 44.6% and 9.6% for EM, IM and PM, respectively, demonstrating a high prevalence of the EM phenotype of the CYP2C19 gene. Reportedly, CYP2C19 gene polymorphisms play a role in Hp eradication using omeprazole-based triple therapy.^17^ Among the CYP2C19 phenotypes, EM has the lowest Hp eradication rate.^18^ The distribution of CYP2C19 gene polymorphisms varies across regions. In China, the prevalence of CYP2C19 gene polymorphisms was reported to be 38.04%, 53% and 8.96% for EM, IM and PM, respectively.^19^

According to the World Health Organization (WHO), Hp-infected individuals exhibit increasing resistance to antibiotics in most regions, with the resistance rate for CAM ≥15%, resulting in a greater risk of treatment failure.^20^ In regions where the resistance rate for CAM is ≥15%, WHO recommends using alternative antibiotics. Given that the local area has a CAM resistance rate of >15%, the drug is not regarded as the first choice for treating children with Hp infection. Considering the high prevalence (45.6%) of the EM phenotype of the CYP2C19 gene in the local area, a comprehensive analysis of CYP2C19 gene polymorphisms (if available) is beneficial for clinicians to use PPIs according to the results.

### Limitations:

There existed a relatively short follow-up period. and failure to exclude and analyse the impact of family socioeconomic factors on the enrolled children. In view of this, further improvements will be made in future research to make more scientific research results.

## CONCLUSIONS

The local area showed a high prevalence of CAM resistance, indicating that CAM is not preferred for Hp eradication. CYP2C19 gene polymorphism analysis exhibited that the EM phenotype is highly prevalent among the population. Therefore, a comprehensive analysis of CYP2C19 gene polymorphisms is recommended to identify ideal PPIs for Hp eradication, thereby improving the eradication rate.

### Authors’ Contributions:

**JZ** and **WY:** Carried out the studies, data collection, drafted the manuscript, and are responsible and accountable for the accuracy or integrity of the work.

**YX** and **XM:** Performed the statistical analysis and participated in its design. Critical review.

**FX:** Literature search, Participated in acquisition, analysis or interpretation of data and drafted the manuscript.

All authors read and approved the final manuscript.
